# Identification of Polymorphisms of the *CSN2* Gene Encoding β-Casein in Greek Local Breeds of Cattle

**DOI:** 10.3390/vetsci8110257

**Published:** 2021-10-30

**Authors:** Dionysios Antonopoulos, Despina Vougiouklaki, George P. Laliotis, Theofania Tsironi, Irene Valasi, Archodoula Chatzilazarou, Panagiotis Halvatsiotis, Dimitra Houhoula

**Affiliations:** 1 Department of Food Science and Technology, Faculty of Food Sciences, University of West Attica, 12243 Athens, Greece; antondion@uniwa.gr (D.A.); dvougiouklaki@uniwa.gr (D.V.); 2 Department of Animal Science, Agricultural University of Athens, 11855 Athens, Greece; glaliotis@aua.gr; 3 Department of Food Science and Human Nutrition, Agricultural University of Athens, 11855 Athens, Greece; ftsironi@aua.gr; 4 Department of Veterinary Science, University of Thessaly, 43100 Karditsa, Greece; evalasi@vet.uth.gr; 5 Department of Wine, Vine and Beverage Sciences, Faculty of Food Sciences, University of West Attica, 12243 Athens, Greece; arhchatz@uniwa.gr; 6 2nd Propaedeutic Department of Internal Medicine, Medical School, National and Kapodistrian University of Athens, “ATTIKON” University Hospital, 12462 Chaidari, Greece; pahalv@gmail.com

**Keywords:** β-casein, b-casomorphin-7, polymorphism, PCR-RFLP

## Abstract

This e research focused on the detection and identification of genetic polymorphisms in exon 7 of the β-casein *CSN2* gene in blood samples from Greek Holstein cows and from local breeds of cattle, such as Vrachykeratiki, Katerinis, and Sykias. For this purpose, DNA was isolated from 780 blood samples obtained from Greek Holstein cows, 86 from three local breeds of cattle, namely Brachyceros, Katerinis, and Sykias, and 14 from Greek buffalo. The desired region of exon 7 was amplified by PCR, resulting in 121 and 251 bp products in bovine and buffalo samples. The PCR product was digested with restriction fragment length polymorphism (RFLP) on agarose gels. The restriction enzymes *DdeI and TaqI* were used. All of the blood samples had the amplified size. The results showed that 74.4% of the Greek Holstein cows had the A2A2 β-casein genotype, the three native breads Vrachykeratiki had 57.7%, and the other two had 100% of the A2A2 β-casein. From the 14 Greek buffalo, 100% had the A2A2 β-casein.

## 1. Introduction

Τhe consumption of cow’s milk protein is responsible for causing allergies in many people. An allergy occurs when a person’s immune system reacts to milk proteins, considering it ‘harmful’, and manifests itself in one or more hypersensitivity reactions [1]. The incidence of cow’s milk protein allergy (especially β-lactoglobulin and β-casein) is estimated to be approximately 10% worldwide [2]. Milk plays an important role in human nutrition and growth, as it is considered an excellent source for the intake of essential amino acids for humans [3]. Whey proteins, apart from providing amino acids, also contribute to the development of intestinal microflora [4]. Bovine milk proteins are divided into two categories: caseins (as1, as2, beta, kappa), which account for 80% of the total proteins, and whey proteins, which make up 20%. Βeta-casein (β-CN) constitutes approximately 35% of the total casein in cow’s milk, has a size of 23,983 kDa, and consists of 209 amino acids in the polypeptide chain. There are 12 variants of β-CN (A1, A2, A3, B, C, D, E, F, G, H1, H2, and I) which are produced in milk, depending on the genotype of each cow [5]. The main types of β-CN are A1 and A2. According to the literature, the production of A1 β-casein is the result of a point mutation in the bovine *CSN2* gene, resulting in the production of A1 β-casein instead of A2 β-casein [6]. The difference between A1 β-casein and A2 β-casein is in an amino acid of the polypeptide chain. A2 β-casein contains proline (CCT) at position 67, while in A1 β-casein, this has been replaced by histidine (CAT) [7]. This amino acid replacement affects the proteolysis of the primary protein structure, leading to the cleavage of different peptides. Decomposition of A1 β-casein leads to the production of an opioid peptide called β-casomorphine-7 (BCM-7), whereas cleavage of A2 leads to the production of β-casomorphine-9 (BCM-9) [3]. BCM-7 is a smaller molecule, which can enter the bloodstream more easily, and has stronger opioid effects. BCM-7 is the substance primarily responsible for gastrointestinal disorders such as bloating, increased gas excretion, and bowel disorders [8].

Currently, three indigenous cattle breeds are mainly bred in Greece, and are considered as threatened with extinction. Two of them, Sykia and Katerini originated from the Primigenius Steppe breed, while the third one, Brachyceros, originated from Bos taurus Brachyceros. Four more autochthonous Greek breeds have been considered as officially extinct (Tinos, Andros, Chios, Kerkyra), one breed as endangered (Kea), while more than 10 unidentified breeds or populations have been reported (Prespa, Rodopi, Crete, Kastelorizo, Nissyros, Lipsi, Agathonissi, Lesvos, Acheloos, Mani). These autochthonous cattle are mainly bred in semi-mountainous and mountainous areas or islands, in free-range farming systems, under poor feeding and lack of management [9,10]. Τhe objective of this study was to identify the frequency and type of genetic polymorphisms in exon 7 of the β-casein CSN2 gene in cows of Holstein, Brachyceros, Katerini, and Sykia breeds, as well as, in Greek buffalo of Greek farms. The results of this study may aid the selection of cattle based on milk quality traits.

## 2. Materials and Methods

### 2.1. Experimental Animals and Farms

#### 2.1.1. Institutional Animal Care and Use Committee (IACUC)

Animal blood sampling was based on the 2010/63 EU guidelines of European Community and Council on the Protection of Animals used for Scientific Purposes; according to Directive Article 1, Paragraph 5, Element F, ‘practices not likely to cause pain, suffering, distress or lasting harm equivalent to, or higher than, that caused by the introduction of a needle in accordance with good veterinary practice’ are permitted for experimental purposes.

#### 2.1.2. Selection and Preparation of Control Samples

Blood samples from Bos taurus, identified as A2A2, A1A2, and A1A1 (characterized as control samples), were acquired directly from the animals, and were kept at −18 °C until the analysis.

#### 2.1.3. Selection and Preparation of Blood Samples

During the study period (September 2020 to May 2021), a total of 780 blood samples were collected from Holstein–Friesian cows from different farms in Greece, and 86 samples were obtained from three native breeds of cattle, namely Vrachykeratiki (*n* = 46), Katerinis (*n* = 20), and Sykias (*n* = 20). In addition, 14 samples were collected from Greek buffalo. The blood samples were kept at −18 °C until the analysis.

#### 2.1.4. Genomic DNA Extraction

DNA was directly extracted from blood samples using an automatic extractor with the Whole Blood Nucleic Acid Extraction Kit, (ZYBIO Company), following the protocol recommended by the supplier. The purity and the quantity of the extracted DNA was evaluated spectrophotometrically by calculating OD_260_/OD_280_ (Epoch spectrophotometer, BioTek, Winooski, VT, USA).

#### 2.1.5. PCR Amplification and Restriction Fragment Length Polymorphism Detection I

After checking for DNA/RNA purity, PCR was carried out. The chosen primers amplify a size of 121 bp of the genomic region of exon 7 of *CSN2*, using 0.6 μM primers forward 5′-CCTTCTTTCCAGGATGAACTCCAGG-3′ and reverse 5′-GAGTAAGAGGAGGG ATGTTTTGTGGGAGGCTCT-3′ [11]. PCR was performed in 50 μL final volume solution using the Master Mix (Hot Start Promega). The amplification was conducted by a thermal cycler (96-well thermal cycler from Applied Biosystems, Thermo Scientific, Agawam, MA, USA), as follows: an initial denaturation at 95 °C for 10 min; followed by 40 cycles with the following step-cycle profile: denaturation at 95 °C for 60 s; annealing at 58 °C for 60 s; extension at 72 °C for 60 s, and a final extension at 72 °C for 10 min.

PCR products were separated in 2% agarose gels, stained with ethidium bromide (0.5 μg/mL), and documented under UV illumination using MiniBIS Pro device (DNR Bio-Imaging Systems Ltd., Neve Yamin, Israel).

**Figure 1:**
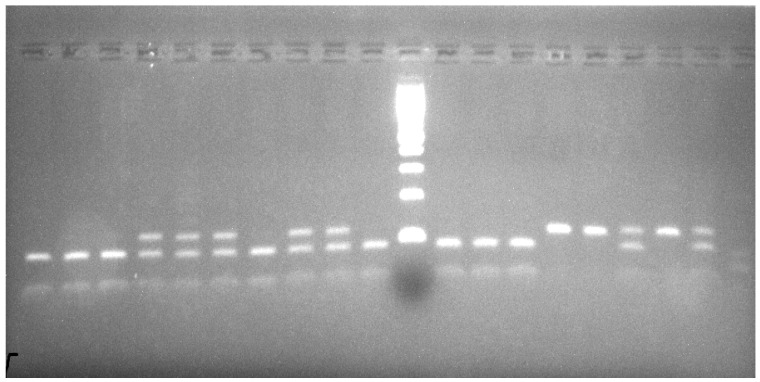
Results of PCR-RFLP analysis for CSN2 gene by DdeI on 3% agarose gel. Lanes 1–3,7,10,12–14 genotype A2A2 (86 and 35 bp), Lanes 4–6,8,9,17,19 genotype A1A2 (121, 86 and 35 bp), Lanes 15,16,18 genotype A1A1 (121 bp), Lane 11 Ladder 100 bp, and Lane 20 Negative control.

The 121 bp PCR products were digested with 5 U of DdeI (Thermo Scientific) restriction enzyme for 1 h at 37 °C. Three types of bands were observed- a complete DdeI cut representing homozygous A2A2 resulting in two fragments (86 and 35 bp); a partial cut representing heterozygous A1A2 resulting in three fragments of 121, 86, and 35 bp; and an uncut 121 bp fragment representing homozygous A1A1 (Figure 1).

#### 2.1.6. PCR Amplification and Restriction Fragment Length Polymorphism Detection II

After checking for DNA/RNA purity, PCR was carried out. The chosen primers amplify a size of 251 bp in the genomic region of exon 7 of *CSN2* using 0.6 μM μL of primers forward 5′-GAGTCGACTGCAGATTTTCAACATCAGTGAGAGTCAGGCCCTG-3′ and reverse 5′-CCTGCAGAATTCTAGTCTATCCCTTCCCTGGGC CCATCG-3′. PCR was performed in 50 μL final volume solution using the Master Mix (Hot Start Promega). The amplification was conducted by a thermal cycler (96-well thermal cycler from Applied Biosystems, Singapore), as follows: an initial denaturation at 95 °C for 10 min; 40 cycles with the following step-cycle profile: denaturation at 95 °C for 60 s; annealing at 65 °C for 60 s; extension at 72 °C for 60 s; and a final extension at 72 °C for 10 min.

PCR products were separated in 2% agarose gels, stained with ethidium bromide (0.5 μg/mL), and documented under UV illumination using MiniBIS Pro device (DNR Bio-Imaging Systems Ltd., Neve Yamin, Israel).

The 251 bp PCR products were digested with 5 U of TaqI (Thermo Scientific, Agawam, MA, USA) restriction enzyme for 1 h at 65 °C. Three types of bands were observed- a complete TaqI cut representing homozygous A1A1 resulting in two fragments (213 and 38 bp); a partial cut representing heterozygous A1A2 resulting in three fragments of 251, 213, and 38 bp; and an uncut 251 bp fragment representing homozygous A2A2.

### 2.2. Statistical Analysis

Analysis of variance (ANOVA) at a significance level of 95% (*p* < 0.05) was used for the evaluation of differences between the observed allele frequencies (STATISTICA 7.0; StatSoft Inc., Tulsa, OK, USA). The allele frequencies were in accordance with the Hardy–Weinberg equilibrium.

## 3. Results

Genomic DNA was isolated from blood samples of Holstein–Friesian cows (*n* = 780) and three native breeds of cattle, Vrachykeratiki (*n* = 46), Katerinis (*n* = 20), and Sykias (*n* = 20), as well as the Greek buffalo (*n* = 14). The extracted DNA was checked for its concentration and purity by photometry. All samples had a concentration of 70–100 ng/μL and a purity of 1.6–1.9 ng/μL. As for the exon 7 of *CSN2* polymorphism, it was shown that the A2 allele occurs with a frequency of 74.4% and 58.7% in Holstein–Friesian cows and Vrachykeratiki, respectively. The A1 allele frequency was 25.6 and 30.4%, respectively. In Katerinis, Sykias, and the Greek buffalo, the A2 allele occurred with a frequency of 100%. In the samples that originated from the Holstein–Friesian breed, 52.2% of them were homozygous for the A2 allele, and 3.3% were homozygous for the A1 allele, while the rest (44.5%) were heterozygous. For Vrachykeratiki breed, the homozygous A2 genotype frequency was 39.1%, while the A1A1 genotype was not observed. Heterozygous genotypes had a frequency of 60.9% (Table 1). The other breeds (Sykias) were 100% homozygous for the A2 allele. There were statistically significant differences between the allele frequency of breeds (*p* < 0.05).

## 4. Discussion

Local bovine breeds can represent a valuable source of genes for selecting certain traits. To our knowledge, the genetic polymorphism of β-casein in cows, indigenous and foreign, that are bred in Greece has not been studied so far.

Improving the knowledge of the genetic diversity within and among local breeds is considered an important issue for enhancing their efficient use for sustainable animal farming in a harsh and less intensified environment, and for implementing further conservation programs [12,13]. As these local breeds are usually unselected and show high adaptability to their local environment, their genetic pool represents a valuable source of genes that may lead to products of high quality [14]. Although local cattle breeds in Greece are considered dual purpose, they are mainly bred for meat purposes rather than for milk production, due to poor infrastructure and supplementary feeding, which lead to a high production cost. Among them, the Vrachykeratiki breed represent the most known local breed, and belongs to the group of Illyrian Brachyceros cattle (*Bos taurus brachyceros*). Katerini and Sykia are two Greek cattle breeds under the threat of extinction that belong to the steppe type of cattle, sharing many similarities with Podolian cattle breeds. Another indigenous bovine breed is the Greek buffalo, which is also under the risk of extinction. The Greek buffalo, which belongs to Murrah type of river buffalo, originates from the Asian buffalo, and is mainly bred for milk and meat. Like all local breeds, they exhibit great adaptability to harsh climate and wetlands, long productive life, low maintenance needs, and production of high-quality products [9,14,15,16]. Investigating their genetic pool to identify polymorphisms that are linked to the production of final products of high-quality traits or traits focusing on the contemporary human needs (i.e., low fat content, enriched in ω3 fatty acids, absence of allergens etc.) is of utmost importance. To our knowledge, the genetic polymorphism of β-casein in cosmopolitan, and especially local bovine, breeds that are bred in Greece has not been thoroughly studied so far. Thus, herein we report the genotyping results of the β-casein locus (*CSN2* gene) in five Greek bovine local breeds and one cosmopolitan breed (Holstein–Friesian) in Greece.

The observed allelic frequencies in the Holstein–Friesian samples are comparable to the respective data in previously reported studies, where a higher frequency of the A2 allele was observed with values ranging from 0.632 to 0.675, similarly to the present study [17,18,19,20].

Another finding emerging from the present study is that A2 allele dominated in the analyzed local bovine breeds, which is in accordance with previous reported studies. Baranyi et al. (1993) reported the high presence of A2 allele of the *CSN2* gene in the Hungarian Gray cattle (pA2 = 0.75). The same trend was also observed for Balkan local breeds such as Busa cattle, Slavonian-Syrmian Podolian cattle, and Istrian cattle [20].

## 5. Conclusions

The results of our study indicate that the β-casein allelic frequencies in the Holstein–Friesian samples were similar to the respective values reported in relevant studies from different geographical regions, while a higher frequency of the A2 allele was observed, with values ranging between 0.632 and 0.675. The A2 allele dominated in the local Greek bovine breeds.

## Figures and Tables

**Table 1:** Gene and genotype frequency of β-casein gene.

Breeds	Total No. of Samples	Genotype Frequency			Allele Frequency	
		A1A1	A1A2	A2A2	A1	A2
Holstein–Friesian	780	(*n* = 26) 3.3%	(*n* = 347) 44.5%	(*n* = 407) 52.2%	25.6%	74.4%
Vrachykeratiki	46	(*n* = 0) 0%	(*n* = 28) 60.9%	(*n* = 18) 39.1%	30.4%	58.7%
Katerinis	20	(*n* = 0) 0%	(*n* = 0) 0%	(*n* = 20) 100%	0%	100%
Sykias	20	(*n* = 0) 0%	(*n* = 0) 0%	(*n* = 20) 100%	0%	100%
Greek buffalo	14	(*n* = 0) 0%	(*n* = 0) 0%	(*n* = 14) 100%	0%	100%

## Data Availability

The data presented in this study are available in the manuscript.
